# Supplementary material for: The Degree of Acceptance of Cocoon Strategy of Vaccination against Influenza and COVID-19 in Palliative Home Care Professionals and Caregivers
Source: Vaccines (Basel). 2023 Jul 12;11(7):1235. doi: 10.3390/vaccines11071235 (PMC10383800; doi:10.3390/vaccines11071235)
Supplement: Supplementary file 1 [file vaccines-11-01235-s001.zip › vaccines-2476946-supplementary.pdf]

Table S1. Questionnaire structure

1	Your role towards the patient [physician; nurse; physiotherapist; psychologist; medical caregiver]
2	Your date of birth [year; month]
3	Your gender [female; male; other]
4	Your main workplace location: rural area; town up to 50,000 inhabitants; city 50,000-100,000 inhabitants; city 100,000-500,000 inhabitants; city >500,000 inhabitants [ordinal scale]
5	Your marital status: [single; couple]
6	Your education: primary, basic vocational, high school, ba, helor's degree, university degree [ordinal scale]
7	Your employment status [studying and working; employed only; working on retirement; pension]
8	What is your main source of information on vaccinations? More than one answer may be given. [medical professional; media; Internet; social portals; acquaintance; family; medical journals; other]; multiple choice
8a	I am not interested in vaccinations [yes; no]
9	How do you assess the health status of your charge? Question for actual caregivers ONLY. [very good; good; average; angry; very angry]
10	Did you have any allergic reactions previously (food, contact, insect venom, inhalants, drugs) [yes; no]
11	Have you suffered COVID-19? [yes; no; do not know]
12	In your immediate vicinity, are there persons who suffered from or are suffering from COVID-19? [yes; no; do not know]
13	In your immediate vicinity, were there persons who died from COVID-19? [yes; no; do not know]
14	Do you follow the recommendations regarding the pandemic:
a	Do you wear a mask when approaching the patient? [yes; no]
b	Do you wear a helmet when approaching the patient? [yes; no]
c	Do you follow the pandemic recommendation to disinfect hands? [yes; no]
d	Do you follow the pandemic recommendation to maintain a social distance? [yes; no]
15	Please rate your attitude to the answers to the following statements:
a	Vaccinations are the most effective way to protect against infectious diseases. [Likert scale]
b	Vaccinations are promoted not because they are really needed but because it is in pharmaceutical companies' interests. [Likert scale]
c	Vaccinations are unnecessary because infectious diseases are rare. [Likert scale]
d	Vaccinations provide more benefits than risks. [Likert scale]
e	I do not need to vaccinate because the risk of getting sick and infecting my charge is small, as I respect the rules of isolation and have no contact with other people. [Likert scale]
16	Do you agree with the following claims about influenza vaccination?

a	Influenza vaccination is safe. [Likert scale]
b	Influenza vaccination is effective. [Likert scale]
c	Influenza vaccination is necessary to avoid illness, complications, and hospitalization. [Likert scale]
d	I will get vaccinated against influenza in order to ensure the epidemiological safety of my patients; charge. [Likert scale]
e	I will get vaccinated against influenza in order to ensure my own epidemiological safety. [Likert scale]
f	Chronic diseases (e.g., cancer, lung and circulatory diseases, diabetes, obesity) cause a severe course of influenza.
g	Influenza is dangerous for my charge; patients. [Likert scale]
h	My knowledge of influenza vaccination is sufficient. [Likert scale]
17	Do you agree with the following claims of vaccination against COVID-19?
a	COVID-19 vaccination is safe. [Likert scale]
b	COVID-19 vaccination is effective. [Likert scale]
c	COVID-19 vaccination is necessary to avoid illness, complications, and hospitalization. [Likert scale]
d	I will get vaccinated against COVID-19 in order to ensure the epidemiological safety of my patients; charge. [Likert scale]
e	I will get vaccinated against COVID-19 in order to ensure my own epidemiological safety. [Likert scale]
f	Chronic diseases (e.g., cancer, lung and circulatory diseases, diabetes, obesity) cause a severe course of COVID-19. [Likert scale]
g	COVID-19 is dangerous for my charge; patients. [Likert scale]
h	My knowledge of COVID-19 vaccination is sufficient. [Likert scale]
18	Do you get vaccinated against influenza? [I have never got vaccinated; Only this season; I got vaccinated but not regularly; Regularly every year]
19	Have you already been vaccinated against COVID-19? [Yes, I have already been vaccinated; No, but I am scheduled for a vaccination; I will not get vaccinated now because I have a high antibody titer after passing COVID-19; I will not get vaccinated because I have contraindications; I will not get vaccinated because I am not sure that the vaccine is safe; I will not get vaccinated because I don't think it's necessary]

Table S2. Opinion on influenza vaccination.

	N	Strongly disagree	Disagree	Neutral	Agree	Strongly agree	Missing
**Influenza vaccination is safe.**							
physician	123	1%	2%	3%	28%	67%	0%
nurse	217	0%	5%	13%	46%	35%	1%
physiotherapist	29	0%	3%	13%	45%	32%	6%
caregiver	41	0%	5%	17%	33%	45%	0%
psychologist	24	0%	4%	13%	46%	38%	0%
**Influenza vaccination is effective.**							
physician	123	1%	1%	7%	50%	41%	1%
nurse	217	0%	7%	23%	43%	25%	1%
physiotherapist	29	0%	10%	13%	58%	16%	3%
caregiver	41	0%	7%	17%	43%	33%	0%
psychologist	24	0%	0%	33%	38%	29%	0%
**Influenza vaccination is necessary to avoid illness, complications, and hospitalization.**							
physician	123	1%	2%	2%	35%	60%	0%
nurse	217	1%	9%	19%	40%	29%	2%
physiotherapist	29	0%	13%	19%	39%	26%	3%
caregiver	41	0%	5%	19%	48%	29%	0%
psychologist	24	0%	4%	33%	25%	38%	0%
**I will get vaccinated against influenza in order to ensure the epidemiological safety of my patients/charge.**							
physician	123	6%	9%	3%	19%	62%	2%
nurse	217	11%	23%	12%	25%	25%	3%
physiotherapist	29	16%	13%	23%	16%	29%	3%
caregiver	41	2%	12%	21%	21%	43%	0%
psychologist	24	0%	33%	17%	4%	42%	4%
**I will get vaccinated against influenza in order to ensure my own epidemiological safety.**							
physician	123	4%	10%	3%	15%	67%	1%
nurse	217	11%	22%	16%	15%	33%	3%
physiotherapist	29	29%	13%	13%	13%	29%	3%
caregiver	41	2%	17%	21%	17%	43%	0%
psychologist	24	0%	33%	17%	4%	42%	4%
**Chronic diseases (e.g., cancer, lung and circulatory diseases, diabetes, obesity) cause a severe course of influenza.**							
physician	123	3%	2%	2%	29%	63%	1%
nurse	217	6%	11%	10%	38%	32%	3%
physiotherapist	29	3%	10%	19%	42%	23%	3%
caregiver	41	2%	14%	33%	17%	33%	0%
psychologist	24	4%	8%	21%	33%	29%	4%
**Influenza is dangerous for my charge/patients.**							
physician	123	2%	2%	3%	27%	61%	5%

nurse	217	1%	4%	8%	47%	37%	3%
physiotherapist	29	3%	6%	13%	32%	39%	6%
caregiver	41	0%	5%	21%	33%	38%	2%
psychologist	24	0%	4%	13%	38%	38%	8%
**My knowledge of influenza vaccination is sufficient.**							
physician	123	1%	6%	4%	41%	47%	2%
nurse	217	0%	7%	11%	56%	24%	2%
physiotherapist	29	0%	6%	10%	55%	23%	6%
caregiver	41	0%	12%	19%	45%	21%	2%
psychologist	24	0%	8%	42%	17%	29%	4%

Table S3. Opinion on COVID-19 vaccination.

	N	Strongly disagree	Disagree	Neutral	Agree	Strongly agree	Missing
**COVID-19 vaccination is safe.**							
physician	123	1%	0%	7%	50%	43%	0%
nurse	217	1%	3%	33%	46%	15%	2%
physiotherapist	29	3%	0%	16%	52%	29%	0%
caregiver	41	2%	0%	29%	38%	26%	5%
psychologist	24	0%	4%	33%	33%	25%	4%
**COVID-19 vaccination is effective.**							
physician	123	1%	0%	15%	46%	38%	1%
nurse	217	0%	3%	44%	39%	12%	2%
physiotherapist	29	3%	0%	19%	58%	19%	0%
caregiver	41	2%	2%	31%	31%	29%	5%
psychologist	24	0%	0%	38%	46%	17%	0%
**COVID-19 vaccination is necessary to avoid illness, complications, and hospitalization.**							
physician	123	1%	0%	3%	22%	74%	0%
nurse	217	1%	4%	18%	44%	32%	1%
physiotherapist	29	3%	0%	10%	52%	35%	0%
caregiver	41	2%	2%	14%	33%	48%	0%
psychologist	24	0%	0%	13%	50%	33%	4%
**I will get vaccinated against COVID-19 in order to ensure the epidemiological safety of my patients/charge.**							
physician	123	2%	2%	3%	7%	85%	2%
nurse	217	5%	6%	9%	28%	50%	2%
physiotherapist	29	6%	3%	3%	23%	58%	6%
caregiver	41	5%	5%	14%	14%	62%	0%
psychologist	24	0%	8%	8%	17%	63%	4%
**I will get vaccinated against COVID-19 in order to ensure my own epidemiological safety.**							
physician	123	2%	2%	2%	6%	89%	0%
nurse	217	3%	6%	10%	26%	53%	2%
physiotherapist	29	6%	3%	3%	29%	58%	0%
caregiver	41	5%	7%	12%	19%	57%	0%
psychologist	24	4%	4%	8%	17%	63%	4%
**Chronic diseases (e.g., cancer, lung and circulatory diseases, diabetes, obesity) cause a severe course of COVID-19.**							
physician	123	2%	0%	2%	20%	75%	1%
nurse	217	4%	6%	10%	28%	50%	2%
physiotherapist	29	6%	10%	10%	26%	48%	0%
caregiver	41	7%	7%	19%	33%	31%	2%
psychologist	24	4%	0%	8%	33%	50%	4%
**COVID-19 is dangerous for my charge/patients.**							
physician	123	1%	0%	2%	15%	80%	2%
nurse	217	0%	2%	10%	30%	54%	3%
physiotherapist	29	0%	0%	6%	32%	58%	3%
caregiver	41	2%	0%	17%	24%	57%	0%
psychologist	24	4%	4%	8%	29%	50%	4%

**My knowledge of COVID-19 vaccination is sufficient.**							
physician	123	1%	5%	11%	43%	40%	1%
nurse	217	3%	14%	16%	44%	21%	2%
physiotherapist	29	0%	0%	23%	58%	16%	3%
caregiver	41	5%	12%	21%	33%	26%	2%
psychologist	24	0%	0%	29%	42%	25%	4%

Table S4. Spearman rank correlation coefficients for general opinion on vaccinations and opinion on influenza and COVID-19. All values are statistically significant (*p* <0.0017—with the Bonferroni correction for multiple comparisons), except for marked with parentheses (*p* <0.05).

	**(1)**	**(2)**	**(3)**	**(4)**	**(5)**	**(6)**
(1) Thanks to vaccinations, many dangerous infectious diseases do not occur today.	1.00	0.62	-0.33	-0.35	0.48	-0.35
(2) Vaccinations are the most effective way to protect against infectious diseases.	0.62	1.00	-0.31	-0.33	0.57	-0.34
(3) Vaccinations are promoted not because they are really needed, but because it is in the interests of pharmaceutical companies.	-0.33	-0.31	1.00	0.57	-0.40	0.55
(4) Vaccinations are unnecessary because infectious diseases are rare.	-0.35	-0.33	0.57	1.00	-0.34	0.66
(5) Vaccinations provide more benefits than risks.	0.48	0.57	-0.40	-0.34	1.00	-0.38
(6) I do not need to vaccinate, because in my case the risk of getting sick and infecting my charge is small, as I respect the rules of isolation and I have no contact with other people.	-0.35	-0.34	0.55	0.66	-0.38	1.00
(7) Influenza vaccination is safe.	0.41	0.47	-0.33	-0.25	0.45	-0.28
(8) Influenza vaccination is effective.	0.35	0.42	-0.26	-0.21	0.45	-0.24
(9) Influenza vaccination is necessary to avoid illness, complications, and hospitalization.	0.36	0.46	-0.27	-0.23	0.43	-0.30
(10) I will get vaccinated against influenza in order to ensure the epidemiological safety of my patients/charge.	0.30	0.40	-0.22	-0.19	0.35	-0.26
(11) I will get vaccinated against influenza in order to ensure my own epidemiological safety.	0.33	0.42	-0.26	-0.22	0.39	-0.27
(12) Chronic diseases (e.g., cancer, lung and circulatory diseases, diabetes, obesity) cause a severe course of influenza.	0.32	0.34	-0.16	-0.19	0.35	-0.22
(13) Influenza is dangerous for my charge/patients.	0.33	0.36	(-0.15)	-0.17	0.32	-0.26
(14) My knowledge of influenza vaccination is sufficient.	0.22	0.29	(-0.12)	(-0.12)	0.30	(-0.14)
(15) COVID-19 vaccination is safe.	0.35	0.46	-0.34	-0.21	0.47	-0.31
(16) COVID-19 vaccination is effective.	0.31	0.43	-0.33	-0.21	0.47	-0.28
(17) COVID-19 vaccination is necessary to avoid illness, complications, and hospitalization.	0.37	0.51	-0.37	-0.26	0.54	-0.38
(18) I will get vaccinated against COVID-19 in order to ensure the epidemiological safety of my patients/charge.	0.39	0.46	-0.36	-0.34	0.51	-0.52
(19) I will get vaccinated against COVID-19 in order to ensure my own epidemiological safety.	0.44	0.53	-0.41	-0.38	0.52	-0.56
(20) Chronic diseases (e.g., cancer, lung and circulatory diseases, diabetes, obesity) cause a severe course of COVID-19.	0.40	0.42	-0.30	-0.27	0.43	-0.34
(21) COVID-19 is dangerous for my charge/patients.	0.39	0.50	-0.30	-0.32	0.45	-0.42
(22) My knowledge of COVID-19 vaccination is sufficient.	0.16	0.30	-0.19	(-0.09)	0.26	-0.16

Table S5. Spearman rank correlation coefficients for opinion on influenza and COVID-19. All values are statistically significant (*p* <0.0017—with the Bonferroni correction for multiple comparisons).

	(1)	(2)	(3)	(4)	(5)	(6)	(7)	(8)	(9)	(10)	(11)	(12)	(13)	(14)	(15)	(16)
(1) Influenza vaccination is safe.	1	0.71	0.7	0.64	0.63	0.39	0.47	0.45	0.58	0.52	0.53	0.49	0.53	0.38	0.41	0.35
(2) Influenza vaccination is effective.	0.71	1	0.78	0.67	0.63	0.42	0.48	0.54	0.6	0.56	0.57	0.5	0.5	0.36	0.43	0.4
(3) Influenza vaccination is necessary to avoid illness, complications, and hospitalization.	0.7	0.78	1	0.72	0.69	0.49	0.53	0.49	0.56	0.54	0.6	0.53	0.55	0.44	0.48	0.39
(4) I will get vaccinated against influenza in order to ensure the epidemiological safety of my patients/charge.	0.64	0.67	0.72	1	0.87	0.41	0.5	0.48	0.52	0.5	0.53	0.56	0.54	0.36	0.39	0.36
(5) I will get vaccinated against influenza in order to ensure my own epidemiological safety.	0.63	0.63	0.69	0.87	1	0.41	0.49	0.45	0.53	0.51	0.54	0.53	0.57	0.38	0.36	0.37
(6) Chronic diseases (e.g., cancer, lung and circulatory diseases, diabetes, obesity) cause a severe course of influenza.	0.39	0.42	0.49	0.41	0.41	1	0.61	0.39	0.35	0.34	0.38	0.35	0.34	0.6	0.46	0.2
(7) Influenza is dangerous for my charge/patients.	0.47	0.48	0.53	0.5	0.49	0.61	1	0.44	0.4	0.37	0.44	0.4	0.39	0.45	0.53	0.25
(8) My knowledge of influenza vaccination is sufficient.	0.45	0.54	0.49	0.48	0.45	0.39	0.44	1	0.4	0.41	0.39	0.31	0.31	0.28	0.33	0.61
(9) COVID-19 vaccination is safe.	0.58	0.6	0.56	0.52	0.53	0.35	0.4	0.4	1	0.8	0.73	0.64	0.63	0.42	0.49	0.53
(10) COVID-19 vaccination is effective.	0.52	0.56	0.54	0.5	0.51	0.34	0.37	0.41	0.8	1	0.73	0.59	0.57	0.44	0.48	0.57
(11) COVID-19 vaccination is necessary to avoid illness, complications, and hospitalization.	0.53	0.57	0.6	0.53	0.54	0.38	0.44	0.39	0.73	0.73	1	0.69	0.68	0.47	0.55	0.5
(12) I will get vaccinated against COVID-19 in order to ensure the epidemiological safety of my patients/charge.	0.49	0.5	0.53	0.56	0.53	0.35	0.4	0.31	0.64	0.59	0.69	1	0.88	0.52	0.62	0.42
(13) I will get vaccinated against COVID-19 in order to ensure my own epidemiological safety.	0.53	0.5	0.55	0.54	0.57	0.34	0.39	0.31	0.63	0.57	0.68	0.88	1	0.51	0.62	0.39

(14) Chronic diseases (e.g., cancer, lung and circulatory diseases, diabetes, obesity) cause a severe course of COVID-19.	0.38	0.36	0.44	0.36	0.38	0.6	0.45	0.28	0.42	0.44	0.47	0.52	0.51	1	0.69	0.31
(15) COVID-19 is dangerous for my charge/patients.	0.41	0.43	0.48	0.39	0.36	0.46	0.53	0.33	0.49	0.48	0.55	0.62	0.62	0.69	1	0.33
(16) My knowledge of COVID-19 vaccination is sufficient.	0.35	0.4	0.39	0.36	0.37	0.2	0.25	0.61	0.53	0.57	0.5	0.42	0.39	0.31	0.33	1

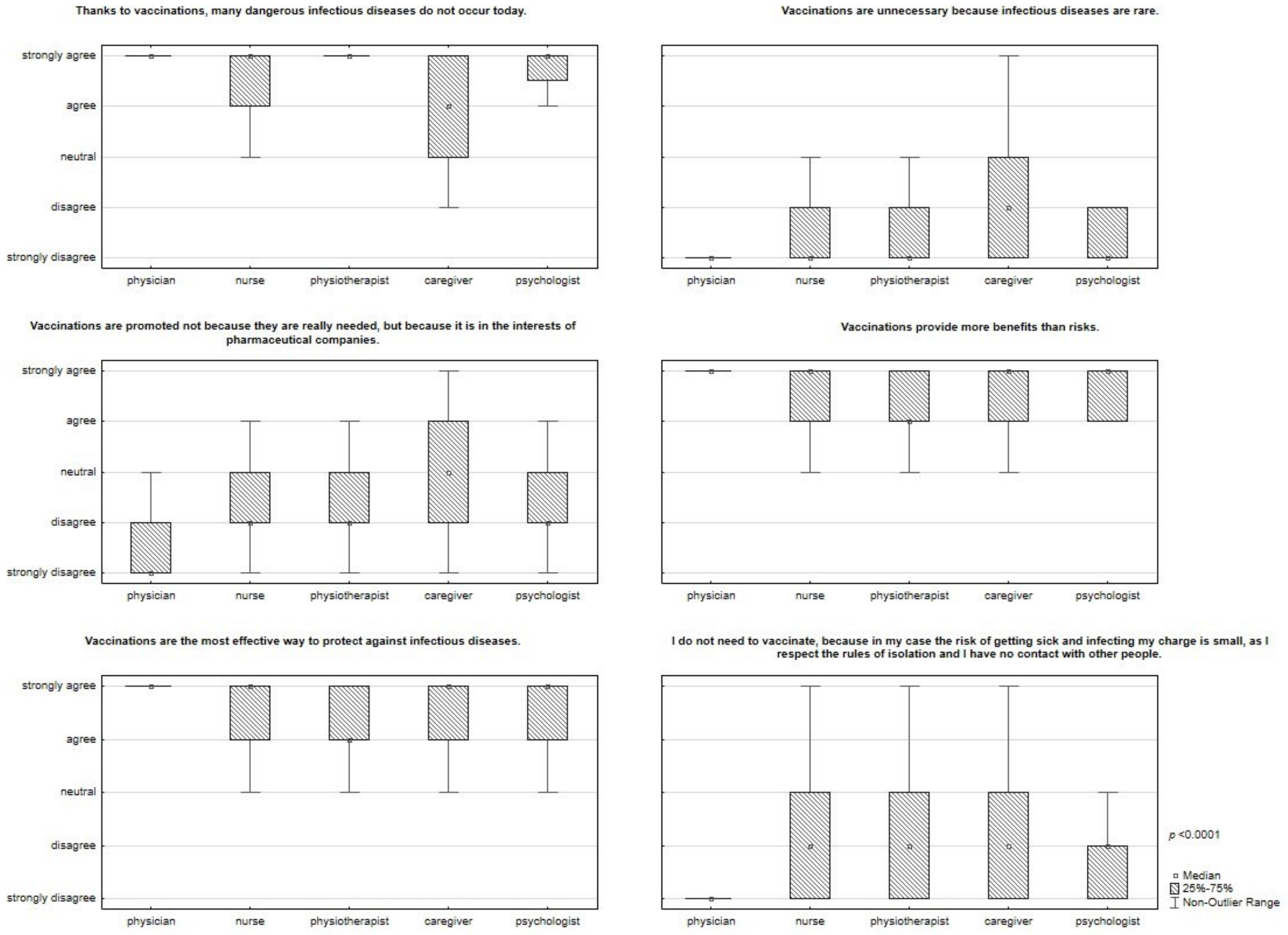

Figure S1. The box-whiskers graph of general opinions on vaccinations in role groups. The differences are statistically significant ($p <0.0001$).
